# Interleukin-18 promotes the antitumor ability of natural killer cells in colorectal cancer via the miR-574-3p/TGF-β1 axis

**DOI:** 10.1080/21655979.2021.1880717

**Published:** 2021-03-04

**Authors:** Yin-Peng Li, Xian-Rong Du, Ru Zhang, Qiu Yang

**Affiliations:** a Department of Gastroenterology, Shenzhen People’s Hospital Longhua Branch (The Second Clinical Medical College, Jinan University; The First Affiliated Hospital, Southern University of Science and Technology), Shenzhen 518020, Guangdong, China; b Department of Gastroenterology, Department of Gastroenterology, Shenzhen People’s Hospital (The Second Clinical Medical College, Jinan University; The First Affiliated Hospital, Southern University of Science and Technology), Shenzhen 518020, Guangdong, China

**Keywords:** IL-18, natural killer cells, tgf-β1, miR-574-3p, microRNA, colorectal cancer

## Abstract

Interleukin (IL)-18 has a clear antitumor effect; however, its mechanisms of action are not understood in patients with colorectal cancer (CRC). Here, we investigated the potential mechanism of IL-18 in CRC. The results showed that IL-18 treatment alone had no effect on HCT116 cells apoptosis, whereas IL-18 in the presence of natural killer (NK) cells resulted in apoptosis and inhibition of cells proliferation in vitro. Profiling of miRNA expression following coculture with NK cells and treatment with IL-18 resulted in significant downregulation of miR-574-3p expression and upregulated expression of the target gene transforming growth factor beta 1 (TGF-β1). miR-574-3p binds to TGF-β1, and miR-574-3p overexpression increased the proliferation and decreased the apoptotic rate of HCT116 cells in NK cells coculture with IL-18 treatment; overexpression of TGF-β1 restored the effect of miR-574-3p overexpression. The miRNA profile of HCT116 undergoes significant alteration before and after coculturing with NK cells and treatment with IL-18. IL-18 alone did not affect HCT116 cells apoptosis but did promote the antitumor ability of NK cells in coculture with HCT116 cells via the miR-574-3p/TGF-β1 axis. Our study suggested that IL-18 can be a new potential target for cancer immunotherapy for CRC.

## GRAPHICAL ABSTRACT

**Figure:**
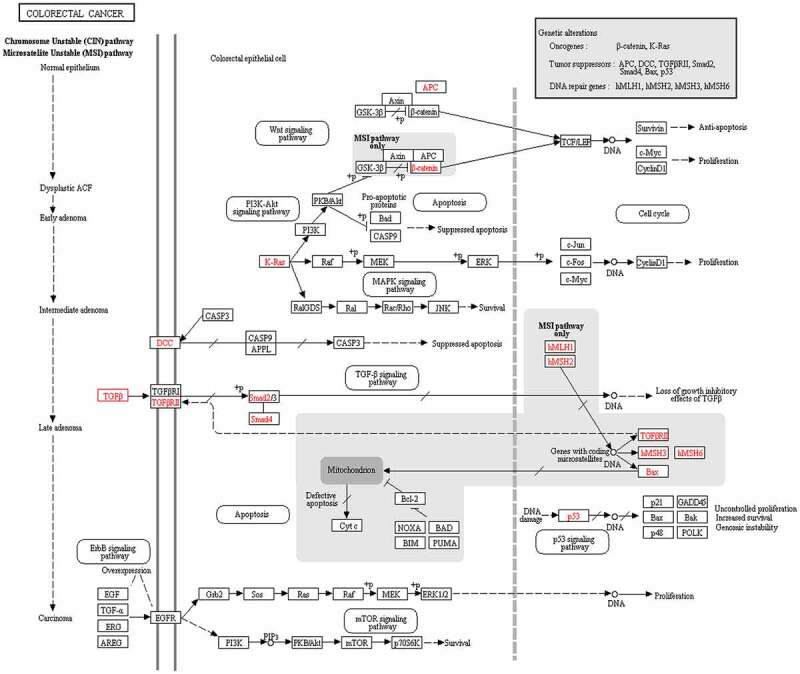

## Introduction

Colorectal cancer (CRC) is one of four most common cancer types globally, with significant mortality and morbidity [1]. Although the incidence of CRC has been decreasing in recent years because of early detection, surgical removal of the tumor area is still the most common treatment of CRC patients [2]. Furthermore, pharmacological options including cytotoxic chemotherapy and antiangiogenic drugs are limited by their toxic side effects. However, as chemotherapy resistance may ultimately occur, there is an urgent need for a novel treatment approach for CRC to provide better healthcare for patients. Immunotherapy has become a rising standard treatment for CRC patients seeking alternative treatment to surgery. Currently, various immune checkpoint blockage drugs are in clinical trials for patients whose CRC is caused by defective mismatch repair (microsatellite instability-high tumors), and this will be extended into the microsatellite stabile population [3]. Nevertheless, it has been difficult to establish an objective response to immunotherapy. Thus, current immunotherapy does not satisfy the clinical need of CRC patients, and there is a need to discover new and more effective immunotherapy targets.

Cytokines have an essential role in the immune system as molecular messengers, allowing communication between immune cells and regulation of immune responses [4]. Interleukin (IL)-18 is a member of the IL-1 superfamily, and there is an increasing recognition in the regulatory role for IL-18 between the innate and acquired immune system [5]. The prognostic value of IL-18 has also been identified in various autoimmune diseases and inflammatory diseases such as rheumatoid arthritis, insulin-dependent diabetes, and multiple sclerosis [3]; the IL-18 precursor is expressed in various cell types throughout the gastrointestinal tract [6]. Combined with the regulatory role of IL-18 in the immune system and a protective role in the gastrointestinal tract, the expression of IL-18 was found to be prognostic in CRC, where a high level of IL-18 is favored (https://www.proteinatlas.org/search/Expression+of+IL18+in+Colorectal+Cancer) [7]. IL-18 has also been indicated to have an important role in other cancer types. For instance, IL-18 was recently found to be expressed by triple-negative breast cancer cells to induce PD-1 expression on immunosuppressive natural killer (NK) cells [8]. IL-18 was also found to establish a pro-inflammatory milieu in the tumor microenvironment of non-small cell lung cancer [9]. IL-18 could also be a potential therapeutic target in multiple myeloma [10], indicating its potential role in cancer development; nevertheless, its role in CRC still needs to be investigated.

Recombinant IL-18 has been shown to have an antitumor function, but this effect has also been indicated with IL-18 receptor (IL-18 R), IL-18 receptor accessory protein (IL-18 RAP), and IL-18 binding protein (IL-18BP). IL-18 R and IL-18 RAP promote the antitumor activity of IL-18, whereas IL-18BP acts as a secreted antagonist that binds and inhibits IL-18 function. Furthermore, recombinant IL-18 can affect antitumor activity by activating NK and T cells [11]; however, some studies have shown that IL-18 can have direct antitumor effects on cells [12].

Cytokines exert CRC processes through the differential regulation of microRNAs (miRNAs) [13]. miRNAs are small noncoding RNAs that can negatively regulate target mRNA post-transcriptionally [14,15]. They are known to participate in the pathogenesis of a broad range of cancers [16,17]. For example, miR-375-3p suppresses the proliferation and metastasis of bladder cancer via target frizzled-8 [18]; miR-96 decreases proliferation, migration, and invasion and can trigger apoptosis in pancreatic cancers [19]; miR-362-3p induces cell cycle arrest through targeting of E2F1, USF2, and PTPN1, which are involved in the recurrence of CRC [20]; and miR-425-5p has been shown to participate in the pathogenesis of KRAS-mutated CRC and thereby increase tumor aggressiveness [21]. miRNA-574-3p has also been reported to play important roles in different cancer types, including prostate cancer, epithelial ovarian cancer, and CRC [22–24]. While the mechanism by which miR-574-3p regulates CRC remains to be investigated, studies have reported that TGF-β1 could significantly induce miR-574-3p upregulation in gastric cancer [25,26].

To explore the antitumor effect of IL-18 in CRC, we first identified a CRC cell lines that exhibited high expression of IL-18 R and IL-18 RAP and low expression of IL-18 BP because this expression pattern usually indicates greater sensitivity to IL-18. Then, HCT116 cells were selected to investigate the antitumor effect of IL-18 with or without coculturing with NK cells. Through miRNA sequencing, bioinformatics analysis, and further validation, we found that miR-574-3p was downregulated and its target mRNA transforming growth factor beta 1 (TGF-β1) was upregulated in IL-18-treated cocultured NK cells. As previous studies have suggested that TGF-β1 significantly induces the upregulation of miR-574-3p in gastric cancer [25,26] but there is no study on CRC, the potential function of miR-574-3p and TGF-β1 in CRC were further studied.

## Material and methods

### Cells culture

Five human colorectal cell lines including SW480, SW620, HCT116, HT-29, and CACO-2 and the human renal epithelial cell line 293 T (Cell Resource Center, Institute of Basic Medical Sciences, Chinese Academy of Medical Sciences, Beijing, China) were cultured in DMEM/F12 with 10% fetal bovine serum (FBS). For passaging, cells were digested with 0.25% trypsin and collected after centrifugation at 1050 × *g* for 5 min. Cells were then passaged at a ratio of 1:3 to 1:6. NK-92® MI cells (ATCC®, CRI 2408^TM^) were cultured in MEMα with 10% horse serum, 10% FBS, 0.1 mM β-mercaptoethanol, and 1% penicillin–streptomycin. For passaging, NK-92® MI cells as a suspension culture were mixed with medium and then were collected by centrifuging as above, followed by passaging at a ratio of 1:2.

### Cells treatment

To select effective IL-18 concentration, HCT116 cells were seeded at 4 × 10^3^ per well (96-well plate) and then were treated with different concentrations of IL-18 (Prospec, Product # CYT-269): 0, 1, and 5 μg/mL. IL-18 was first prepared in 0.1% bovine serum albumin and then added into the media accordingly. To select treatment time, HCT116 cells were tested at five different timepoints: 0, 24, 48, 72, and 96 h. HCT116 cells were indirectly cocultured with NK-92® MI cells (NK) at a ratio of 1:3. For cells transfection, HCT116 cells or 293 T cells were seeded into wells, transfected using Lipo 3000 (ThermoFisher, Product # L3000-015), and then used for further experiments after 48 h incubation.

### Plasmid construction and mimic synthesis

For overexpression of TGF-β1, the coding domain sequence was cloned into LV003 vector and was then transfected into HCT116; TGF-β1 expression was validated by reverse transcriptase quantitative polymerase chain reaction (RT-qPCR). For detection of miR-574-3p and TGF-β1 3′UTR (untraslated regions) binding, the TGF-β1 wild-type (Wt) and mutant type (Mut) 3′UTR were prepared by first analyzing the sequence of miR-574-3p and TGF-β1 3′UTR and then finding the possible binding sites of miR-574-3p and TGF-β1 3′UTR using miRTarBase (http://mirtarbase.mbc.nctu.edu.tw/php/detail.php?mirtid=MIRT733732#target); the designed sequence was synthesized by General Biosystems (Anhui, China) and cloned into pmirGL0 (Promega, Beijing, China) to give pmirGL0-Wt and pmirGL0-Mut. For overexpression of miR-574-3p, miR-574-3p mimics and negative control (NC) mimics were synthesized by GenePharma (Shanghai, China) and were subsequently transfected into HCT116; expression was validated by RT-qPCR.

### RT-qPCR

RT-qPCR was used to measure the expression level of IL-18 R, IL-18 RAP, and IL-18BP in the five CRC cell lines, the expression level of TGFBR1, TGFB1, and IL-17A in HCT116 after treatment, and the expression of miRNAs. RNA was extracted using TRIzol (MRC, TR118-500), and cDNA was synthesized with M-MLV reverse transcriptase (Promega, M1705). qPCR was conducted using the ChamQ SYBR qPCR Master Mix (Vazyme, Q341-03) using the primers in Table 1 on a StepOnePlus^TM^ Real-Time PCR system (Applied Biosystems). Target gene expression was normalized to expression of GAPDH. Similarly, the relative miRNA expression including miR615-5p, miR-7-1-3p, let-7 c-5p, miR-574-3p, and miR-510-3p was assessed using the primers in Table 1. Expression of miRNA was normalized to expression of U6.

**Table 1.** Sequences of primers used for quantitative qPCR assay

Name	Sequence (5ʹ-3ʹ)
IL-18R-F	CTTACCTAAAAGAATGCCGACC
IL-18R-R	CAACACCCTGGGCAAAATCT
IL-18RAP-F	AGCCCCTATGATGTAGCCTGTT
IL-18RAP-R	TTACCGCTGGACTTTGTGCA
IL-18BP-F	TGCCACTGAATGGAACGCTG
IL-18BP-R	TGGGAGGTGCTCAATGAAGGA
GAPDH-F	GAGTCAACGGATTTGGTCGT
GAPDH-R	GACAAGCTTCCCGTTCTCAG
TGFBR1-F	CACAGAGTGGGAACAAAAAGGT
TGFBR1-R	CCAATGGAACATCGTCGAGC
TGF-β1-F	TAAAAGTGGAGCAGCACGTG
TGF-β1-R	GTGAACCCGTTGATGTCCAC
IL17A-F	GACTCCTGGGAAGACCTCATTG
IL17A-R	CCGGTTATGGATGTTCAGGTTG
miR-615-5p-RT	GTCGTATCCAGTGCAGGGTCCGAGGTATTCGCACTGGATACGACGATCCG
miR-615-5p-F	GGGTCCCCGGTGCTCG
hsa-miR-7-1-3p-RT	GTCGTATCCAGTGCAGGGTCCGAGGTATTCGCACTGGATACGACTATGGC
hsa-miR-7-1-3p-F	GCTCAACAAATCACAGTCTGC
hsa-let-7 c-5p-RT	GTCGTATCCAGTGCAGGGTCCGAGGTATTCGCACTGGATACGACAACCAT
hsa-let-7 c-5p-F	GCGCTGAGGTAGTAGGTTGTAT
hsa-miR-574-3p-RT	GTCGTATCCAGTGCAGGGTCCGAGGTATTCGCACTGGATACGACTGTGGG
hsa-miR-574-3p-F	GCGCCACGCTCATGCACACACC
hsa-miR-510-3p-RT	GTCGTATCCAGTGCAGGGTCCGAGGTATTCGCACTGGATACGACTCCACT
hsa-miR-510-3p-F	GCGCATTGAAACCTCTAAGAG
UNIVERSE-R	GTGCAGGGTCCGAGGT
U6-F	CGCTTCGGCAGCACATATAC

### Cells proliferation assay

CellTiter 96® Aqueous One Solution Cell Proliferation Assay (Promega, Catalog # G3582) was used to measure treated HCT116 cells proliferation.

### Cells apoptosis assay

The rate of apoptosis of HCT116 following treatment was measured using allophycocyanin (APC)-Annexin V staining (550474, BD PharmingenTM) and 7-aminoactinomycin D (7-AAD, 559925, BD Pharmingen). Cells were collected after digestion with ethylene diamine tetraacetic acid (EDTA)-free trypsin and were resuspended at 1 × 10^6^ cells/mL. Cells solution (100 μL) was stained with 5 μL of annexin V and 5 μL of 7-AAD. After incubating in the dark for 15 min, cells were assessed within 1 h using an Accuri C6 flow cytometer (BD).

### miRNA sequencing and analysis

RNA of HCT116 cells after treatment was extracted as described above. RNA concentration and purity were assessed via Nanopro 2010 (ThermoFisher, Waltham, MA, USA). RNA of 18–30 nt was separated using polyacrylamide gel electrophoresis (PAGE) gels. Separated small RNA was first bound to 3′ primers and then centrifuged and bound to 5′ primers. Small RNAs were then amplified using PCR and further purified using PAGE gels. The collected bank of small RNAs was sequenced by Novogen Co., Ltd (Beijing, China).

The expression of miRNA was then analyzed. Changes in miRNA expression were considered significant when a 1.5-fold change occurred and the Q value (or false discovery rate, FDR) was less than 0.05. To identify the functions of the miRNAs of interest, miRNA related genes of interest were assessed by BLAST searches in the Gene Ontology (GO) database (http://www.geneontology.org) and the Kyoto Encyclopedia of Genes and Genomes (KEGG) database (https://www.genome.jp/kegg/).

### GO and KEGG pathway analysis

The Database for Annotation, Visualization and Integrated Discovery (http://david.abcc.ncifcrf.gov/) was used to annotate the potential functions of the differentially expressed target genes of miRNAs in various signaling pathways. First, they were screened by the selection criteria of fold change ≥ 2 and *p*-value< 0.05. Then, the biological function and pathway of the overlapping genes were enriched in GO and KEGG pathways.

### Dual-luciferase assay

293 T cells were seeded in 24-well plates and divided into six groups: pmirGL0-Wt (cotransfected with pmirGL0-Wt and NC mimics), pmirGL0-Wt + mimics (cotransfected with pmirGL0-Wt and miR-574-3p mimics), pmirGL0-Mut (cotransfected with pmirGL0-Mut and NC mimics), pmirGL0-Mut + mimics (cotransfected with pmirGL0-Mut and miR-574-3p mimics), pmirGL0 (cotransfected with pmirGL0 and NC mimics), and pmirGL0 (cotransfected with pmirGL0 and miR-574-3p mimics). Luciferase activity was detected 48 h after cotransfection using the Dual-Luciferase Reporter Gene Assay Kit (Beyotime, Catalog # RG027). Luciferase activity was measured on a multi-label microplate reader (Tecan Spark). Bioluminescent signals were quantified based on firefly fluorescence value/Renilla fluorescence value.

### Statistical analysis

Data (mean ± standard deviation, SD) and figures were analyzed with GraphPad Prism7.0 (GraphPad software, CA, USA, www.graphpad.com) and SPSS 10.0 (IBM, Armonk, NY, USA). T-test and one-way analysis of variance was performed to assess the data.

## Results

HCT116 cells exhibited high expression of IL-18 R and IL-18 RAP but low expression of IL-18BP, indicating good functional activity of IL-18; therefore, HCT116 was used for further experiments to determine the effects of exogenous IL-18 on CRC. IL-18 treatment alone did not affect cells apoptosis but it enhanced the antitumor ability of NK cells. Then, we collected and sequenced miRNAs to identify the underlying mechanism and further selected and verified several miRNAs. We found that miR-574-3p may play an essential role in the antitumor abilities of IL-18-induced NK cells, probably by directly binding to TGF-β1. Finally, the interaction between TGF-β1 and miR-574-3p and the mechanism involved in regulating CRC were studied.

### Exogenous IL-18 had no effect on CRC but promoted the killing effect of NK cells on CRC

**Figure 1.**
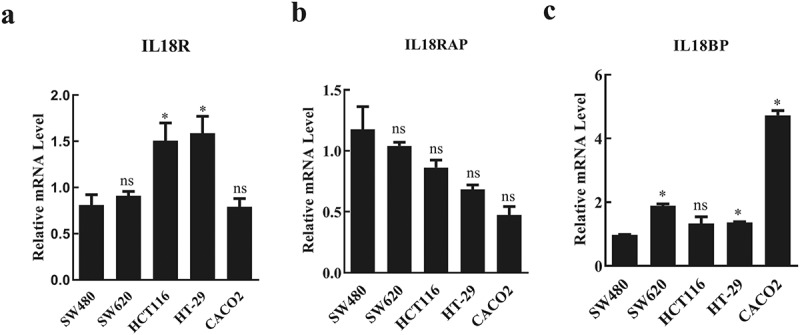
Expression levels of IL-18R, IL-18RAP, and IL-18BP in different colorectal cancer cell lines

Functional activity of IL-18 is dependent on binding to IL-18 R. However, two other proteins affect this process: IL-18BP, which binds to IL-18 and inhibits the binding of IL-18 to IL-18 R, and IL-18 RAP, which promotes the binding of IL-18 to IL-18 R. To determine which CRC cell lines have high expression of IL-18 R and IL-18 RAP and low expression of IL-18BP, we tested the expression level of these genes in five different colorectal cell lines (SW480, SW620, HCT116, HT-29, and CACO2) using RT-qPCR. Among these cell lines, HCT116 and HT-29 had significantly higher expressions of IL-18R compared with that in SW480, SW620, and CACO2 cell lines (Figure 1a). In comparison with HT-29 cells, HCT116 cells exhibited a higher expression of IL-18RAP, although there was no significant difference in IL-18BP expression (Figure 1b,c). Therefore, HCT116 was used for further experiments to determine the effects of exogenous IL-18 on CRC.

a. Relative mRNA levels of IL-18R in colorectal cancer cell lines. HTC116 and HT-29 cells had significantly higher expression levels of IL-18R compared with expression in SW480, SW620, and CACO2 cell lines. b. Relative mRNA levels of IL-18RAP in colorectal cancer cell lines. IL-18RAP expression was higher in HCT116 cells compared with that in HT29 cells. c. Relative mRNA levels of IL-18BP in colorectal cancer cell lines. Both HCT116 and HT-29 had relative low expressions of IL-18BP. IL-18R: IL-18 receptor; IL-18RAP: IL-18 receptor accessory protein; IL-18BP: IL-18 binding protein. All data with error bars are presented as mean + SD, One-way analysis: * *p* < 0.05, compared with SW480, ns: not significant compared with SW480.

**Figure 2.**
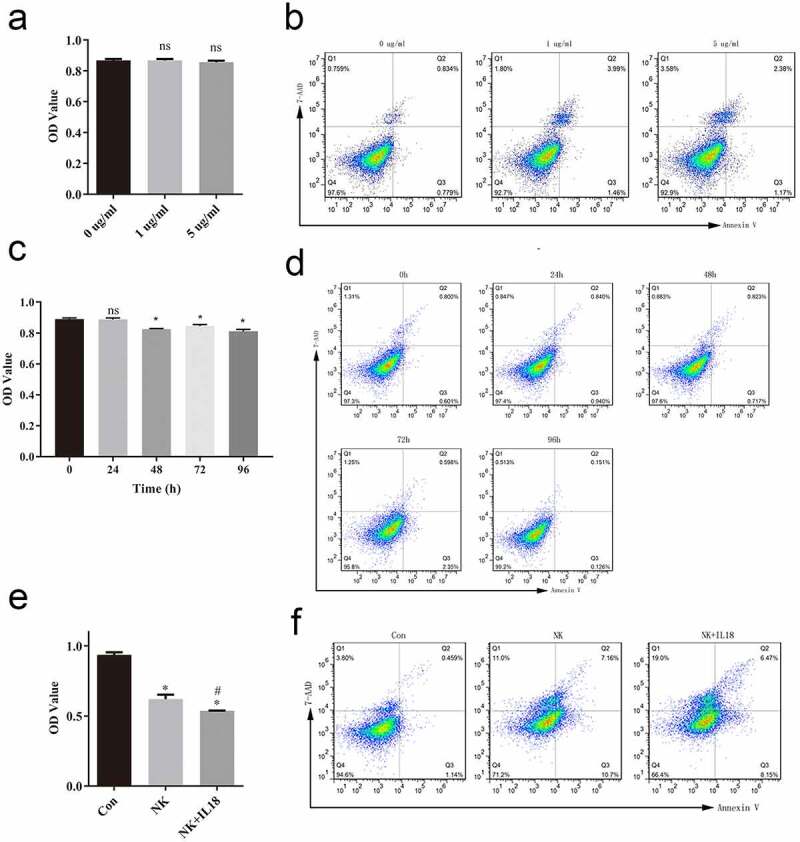
IL-18 treatment had no effect on HCT116 cells but improved the antitumor effect of NK cells

Exogenous IL-18 was added to treat HCT116 cells at 1 and 5 μg/mL, using no treatment as control. Cells proliferation was measured using MTS after 48 h treatment. There was no significant change in the rate of cells proliferation between 1 and 5 μg/mL groups and control group although cells proliferation decreased a little, whereas cells apoptosis decreased in the 1 μg/mL and 5 μg/mL groups compared to control group, although there is no significant difference (Figure 2a,b). To observe the time duration of IL-18 in HCT116, cells were treated with 5 μg/mL IL-18 and tested at 0, 24, 48, 72, and 96 h post-treatment. IL-18 treatment affect the rate of cells proliferation at and 48, 72, and 96 h, whereas there is no difference in cells apoptosis. (Figure 2c,d). As the difference beween groups were not enlarge after 5 μg/mL IL-18 treatment for 96 h, we thought the above results indicated that exogenous IL-18 may have no effect on CRC proliferation and apoptosis. Therefore, we suspected that exogenous IL-18 has an effect on HCT116 cells via NK or T cells. To test this hypothesis, we cocultured HCT116 cells with NK cells with 5 μg/mL of IL-18 treatment for 48 h. The presence of NK cells alone reduced HCT116 cells proliferation compared with that of the control group (HCT116 cells only). Addition of IL-18 (NK+IL-18, HCT116 coculture with NK cells under IL-18 treatment) significantly enhanced the antitumor ability of NK cells compared with that of the NK group (NK cells and HCT116 cocultured with NK cells) (Figure 2e). Similarly, the percentage of 7-AAD-positive and annexin V-positive cells was increased in the presence of NK cells and was further increased when IL-18 was present (Figure 2f), indicating that IL-18 enhanced the antitumor capability of NK cells by inducing more apoptosis in HCT116 cells.

a. Cells proliferation of different IL-18 dosing groups. b. Representative fluorescence-activated cells sorting of 7-AAD-positive and annexin V-positive cells at different doses. The increase of IL-18 dose results in non-significant increases in abundance of 7-AAD-positive and annexin V-positive cells. c. Cells proliferation at different time points during IL-18 treatment. d. Representative fluorescence-activated cells sorting of 7-AAD-positive and annexin V-positive cells at different timepoints. e. MTS assay of HCT116 cells proliferation cocultured with NK cells only or NK cells and IL-18 treatment. f. Flow cytometry assessment of 7-AAD- and annexin V-positive cells. IL-18: Interleukin 18; NK cells: Natural killer cells; MTS: 3-(4,5-Dimethylthiazol-2-yl)-5-(3-carboxymethoxyphenyl)-2-(4-sulfophenyl)-2 H-tetrazolium, Con: control. All data with error bars are presented as mean + SD, One-way analysis: * *p* < 0.05, compared with 0 group or control group; ns: not significant, compared with 0 group or control group; #*p* < 0.05, compared with NK group.

### NK cells and IL-18 treatment altered the expression of miRNA involved in apoptosis and cells proliferation in HCT116 cells

**Figure 3.**
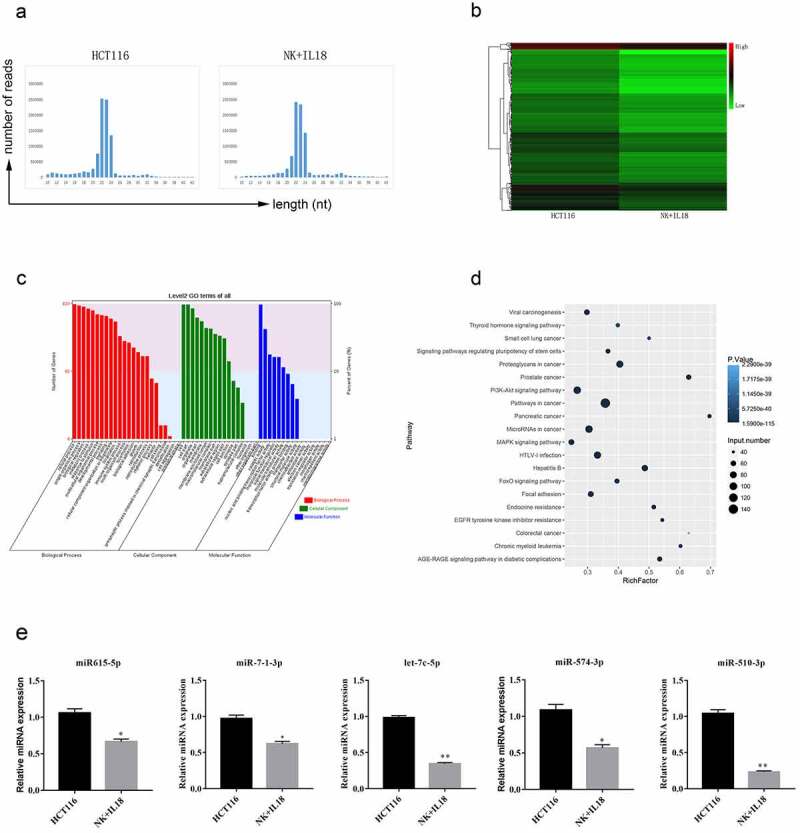
miRNA sequencing from HCT116 cells before and after NK cells coculturing and IL-18 treatment

To better identify the underlying mechanism by which IL-18 and NK cells affect HCT116 cells proliferation and apoptosis, we collected and sequenced miRNA from HCT116 cells before and after coculturing with NK cells and IL-18 treatment. The majority of sequenced RNAs from both samples were between 21 to 24 nucleotides (nt) in length as expected (Figure 3a). Sequences outside this range were not considered miRNA and therefore not included in future studies. Within these sequenced RNAs, the expressions of each miRNA were compared before and after HCT116 coculturing with NK cells and treatment with IL-18. On the basis of a 1.5-fold change, we found 12 significantly upregulated miRNAs and 697 significantly downregulated miRNAs (Figure 3b, *p*-value ≤ 0.05). To elucidate the function of each miRNA, we conducted GO analysis among the target genes of the differentially expressed miRNAs and categorized the miRNAs into three different functional groups: biological processes (including cellular process, biological regulation, signaling, metabolic process, and developmental process), cellular compartments (including organelle, membrane, extracellular region, cell junction, and synapses), and molecular functions (including catalytic activity, signal transducer activity, binding, and protein activity). We found that majority of the differentially expressed miRNA were involved in biological processes (Figure 3c), with the highest number of counts in cellular process, single-organism process, biological regulation, and metabolic process. To confirm the biological relevance of these miRNAs, we conducted a KEGG pathway enrichment analysis. Target genes of these miRNAs were mainly involved in pathways in cancer, PI3K-AKT signaling pathways, and microRNAs in cancer (Figure 3d).

a. Length of sequenced RNAs before and after NK cells coculturing and IL-18 treatment. miRNAs from both groups were distributed between 21 and 24 nt in length. b. Heatmap of differentially expressed miRNA. Twelve miRNAs had increased expression after treatment, whereas 697 miRNAs had decreased expression after treatment. (Difference in expression level is equal or greater than 1.5-fold was required to be considered as a change in expression.) c. Gene Ontology analysis of target mRNA of differentially expressed miRNAs. The majority of differentially expressed miRNAs are involved in biological processes. X-axis, biological pathways grouped as biological processes (red), cellular compartments (green), and molecular functions (blue); y-axis, count of miRNAs in each pathway. d. Kyoto Encyclopedia of Genes and Genomes pathway enrichment analysis. Target genes of the differentially expressed miRNAs are mainly involved in cancer, PI3K-AKT signaling, and function of microRNAs in cancer. e. Expression verification of hsa-miR615-5p, hsa-miR-7-1-3p, hsa-let-7c-5p, hsa-miR-574-3p, and hsa-miR-510-3p using qPCR. NK: Natural killer; IL-18: Interleukin 18; PI3K-AKT: Phosphatidylinositol 3-kinase and protein kinase B; qPCR: Quantitative polymerase chain reaction. All data with error bars are presented as mean ± SD, Student’s t test: * *p* < 0.05, ** *p* < 0.01.

On the basis of the above categorization of the sequenced miRNAs, we selected five different miRNAs of interest for further experiments. The selected miRNAs showed changes in expression before and after coculturing of HCT116 cells with NK cells and treatment with IL-18 and have been previously reported to have important functions in cells proliferation and apoptosis [27–30]. Thus, we focused our future studies on hsa-miR615-5p, hsa-miR-7-1-3p, hsa-let-7c-5p, hsa-miR-574-3p, and hsa-miR-510-3p, as they displayed regulatory functions on proteins including IGF2, RAB4, EGFR, IGF1R, TGFBR1, MYC, and SMAD4 (Table 2). We first verified changes in the expression of these miRNAs via RT-qPCR. These five miRNAs were significantly downregulated after HCT116 coculture with NK cells and treatment with IL-18 (Figure 3e), confirming the results of sequencing.

**Table 2.** miRNA selection and expression verification

miRNA ID	HCT116-TPM	IL_18-TPM	log2 Ratio (IL_18/HCT166)	Up-Down-Regulation (IL_18/HCT166)	P-value	FDR	Target Genes
miR-615-5p	130.3934	37.5106	−1.7975	Down	0	2.29E-104	IGF2, RAB24
miR-7-1-3p	1601.105	927.1425	−0.7882	Down	0	0	EGFR, IGF1R, RAF1, KCNJ2
let-7c-5p	343.9652	220.3056	−0.64276	Down	0	3.95E-49	CDC25A, TGFBR1, MYC
miR-574-3p	89.27742	57.75968	−0.62823	Down	0	1.30E-12	EGFR, SMAD4, TGFB1
miR-510-3p	80.33917	23.23665	−1.7897	Down	0	5.64E-64	IL-17A, IL-17, ZNF790

### miR-574-3p regulated TGF-β1 expression in the NK + IL-18 group

**Figure 4.**
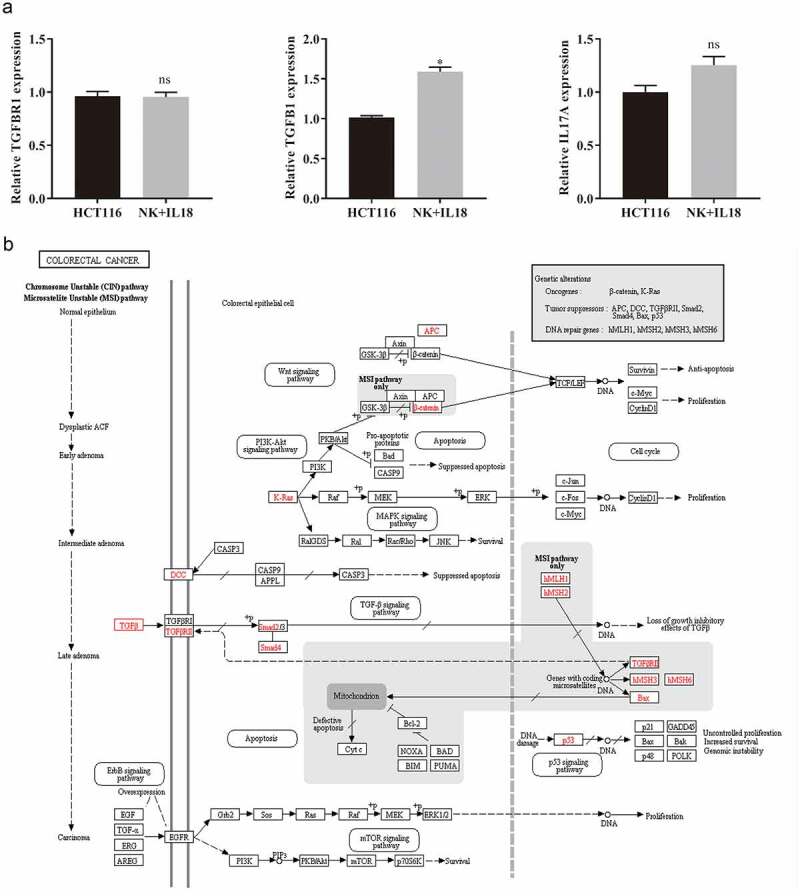
miRNA targets and pathways of TGF-β1 in colorectal cancer

To identify miRNAs that affect cells proliferation and apoptosis, we focused on three miRNAs, let-7c-5p, miR-574-3p, and miR-510-3p, which had the greatest changes in the expression levels in the NK + IL-18 group compared with that in HCT116 cells only. These three miRNAs were previously reported to regulate gene expressions in apoptotic and/or pro-inflammatory pathways [27,29,30]. Specifically, let-7c-5p regulates transforming growth factor, beta receptor 1 (TGFBR1); miR-574-3p regulates TGF-β1; and miR-510-3p regulates IL-17A. We found that the expression of TGFBR1 and IL-17A remained unchanged in the NK + IL-18 group compared with expression in the HCT116 cells only group, whereas the expression of TGF-β1 significantly increased in HCT116 cells (Figure 4a, p < 0.05). KEGG pathway analysis in CRC demonstrated that TGF-β is involved in cells proliferation and apoptosis (Figure 4b). Therefore, we hypothesized that miR-574-3p may play an essential role in the antitumor activities with NK + IL-18 treatment via TGF-β1; TGF-β 1 is a member of the TGF-β family.

a. Target gene expressions of hsa-let-7 c-5p, hsa-miR-574-3p, and hsa-miR-510-3p were verified by qPCR. b. Pathways of TGF-β1 in colorectal cancer. qPCR: Quantitative polymerase chain reaction; TGF-β1: Transforming growth factor beta 1. All data with error bars are presented as mean + SD, Student’s t test: * *p* < 0.05, ns: not significant.

**Figure 5.**
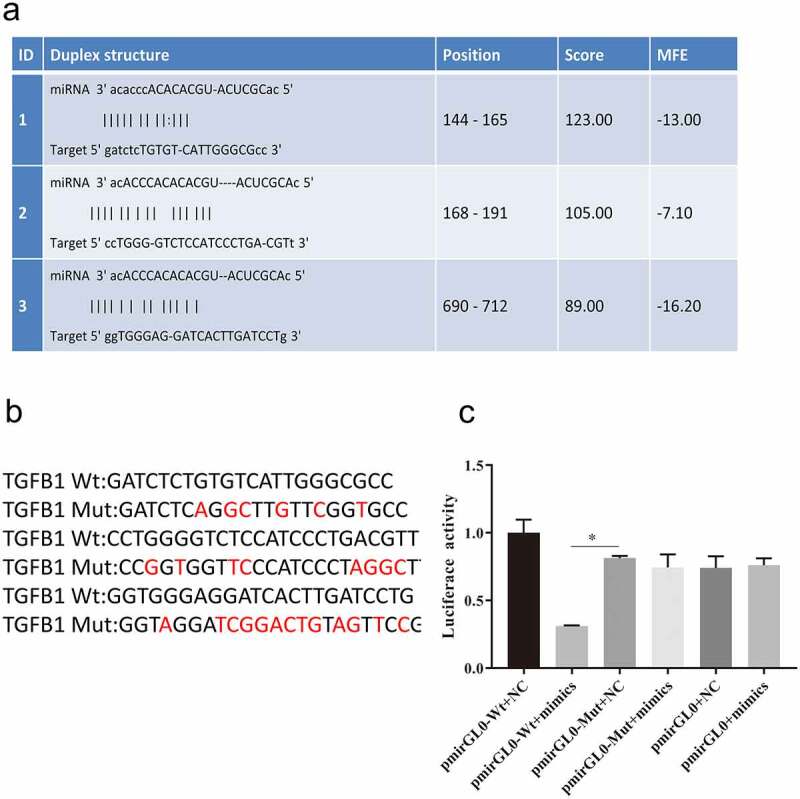
miR-574-3p binding to TGF-β1

To elucidate the relationship between TGF-β1 and miR-574-3p, a dual-luciferase reporter assay was used to study the interaction between TGF-β1 and miR-574-3p. The binding site of TGF-β1 and miR-574-3p was predicted using miRTarBase (http://mirtarbase.mbc.nctu.edu.tw/php/index.php) (Figure 5a). There are three potential direct binding sites of miR-574-3p on TGF-β1. To verify the binding, we created a mutant TGF-β1 containing all three mutations (Figure 5b). Wild-type TGF-β1 (pmirGL0-Wt) and mutant TGF-β1 (pmirGL0-Mut) were individually contransfected with miR-574-3p mimics or NC mimics into 293T cells. Cotransfection of pmirGL0-Wt and miR-574-3p mimics significantly decreased the luciferase activity compared with that of the mutated TGF-β1 group (Figure 5c). This result confirmed the inhibitory effect of miR-574-3p on TGF-β1; such regulation is possibly accomplished via direct binding between miR-574-3p and TGF-β1.

a. Predicted binding sites of miR-574-3p and TGF-β1. b. Induced mutations in TGF-β1 gene. Red locus indicated the specific induced mutations. c. Luciferase activity assays were significantly reduced when miR-574-3p was overexpressed and were restored when TGF-β1 was mutated. TGF- β1: Transforming growth factor beta 1. All data with error bars are presented as mean + SD, One-way analysis: * *p* < 0.05.

### Overexpression of miR-574-3p inhibits the antitumor effect of NK cells and IL-18 treatment, whereas overexpression of TGF-β1 rescued the effect of NK cells and IL-18

**Figure 6.**
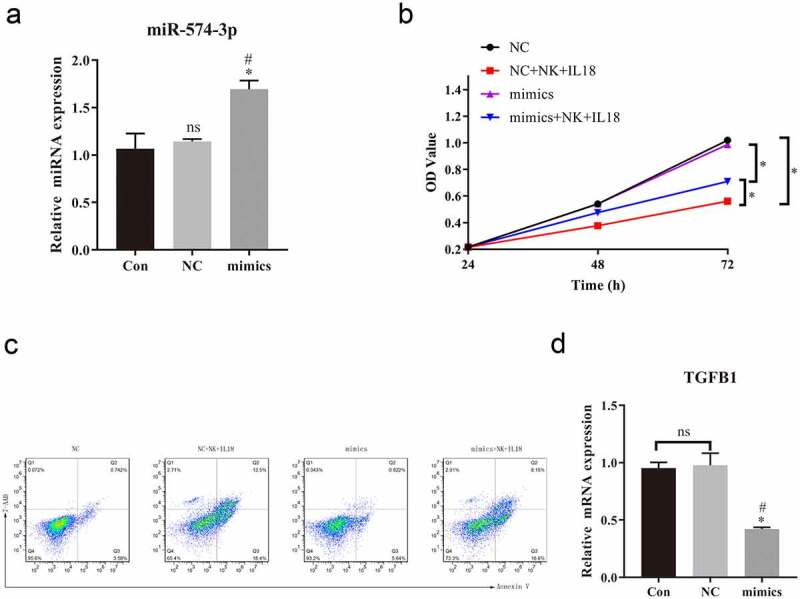
Overexpression of miR-574-3p diminished the antitumor effects of NK cells and IL-18

To evaluate the effects of miR-574-3p on cells proliferation and apoptosis, miR-574-3p mimics were transfected into HCT116 cells. miR-574-3p expression increased after transfection with miR-574-3p mimics compared with expression in HCT116 cells only and in cells transfected with miR-574-3p NC (Figure 6a). Transfection with miR-574-3p mimics did not change the proliferation rate and apoptosis rate of HCT116 cells compared with that of the mimics NC (Figure 6b,c). However, when HCT116 was cocultured with NK cells and then treated with IL-18, the rate of cells proliferation decreased and rate of apoptosis increased (Figure 6b,c). Interestingly, cells proliferation was significantly restored in HCT116 cells when miR-574-3p was overexpressed when compared with proliferation of HCT116 cells with NC mimics during NK cells coculturing and IL-18 treatment. Similarly, when HCT116 cells overexpressed miR-574-3p, a lower level of apoptosis was found compared with expression in control cells. Our results indicate that miR-574-3p can inhibit the antitumor effects of NK cells and IL-18 by restoring cells proliferation and reducing apoptosis. Furthermore, we also found that the expression of TGF-β1 was significantly reduced when miR-574-3p was overexpressed (Figure 6d). The above results indicated that miR-574-3p inhibits the effects of NK cells and IL-18, possibly by downregulation TGF-β1 expression; thus, overexpression of TGF-β1 may promote the role of NK cells and IL-18 and reverse the function of miR-574-3p.

a. qPCR was used to assess expression of miR-574-3p. Increased expression of miR-574-3p after transient transfection of miR-574-3p mimics. b. MTS assay showing that miR-574-3p mimics partially restored the cells proliferation rate of HCT116 with NK cells coculturing and IL-18 treatment. c. Representative fluorescence-activated cells sorting of 7-AAD positive and annexin V-positive cells of HCT116 when miR-574-3p was expressed. The overexpression of miR-574-3p alone did not change the apoptotic rate of HCT116 but rescued the HCT116 from apoptosis with NK cells coculturing and IL-18 treatment. d. qPCR results indicated that overexpression of miR-574-3p significantly reduced the expression of TGF-β1. NK: Natural killer; IL-18: Interleukin 18; MTS: 3-(4,5-Dimethylthiazol-2-yl)-5-(3-carboxymethoxyphenyl)-2-(4-sulfophenyl)-2 H-tetrazolium; qPCR: Quantitative polymerase chain reaction; TGF-β1: Transforming growth factor beta 1, con: control grouop; NC: negative control of miR-574-3p mimics group. All data with error bars are presented as mean + SD, One-way analysis: * *p* < 0.05, compared with Con groupns; # *p* < 0.05, compared with NC groupns; ns: not significant.

**Figure 7.**
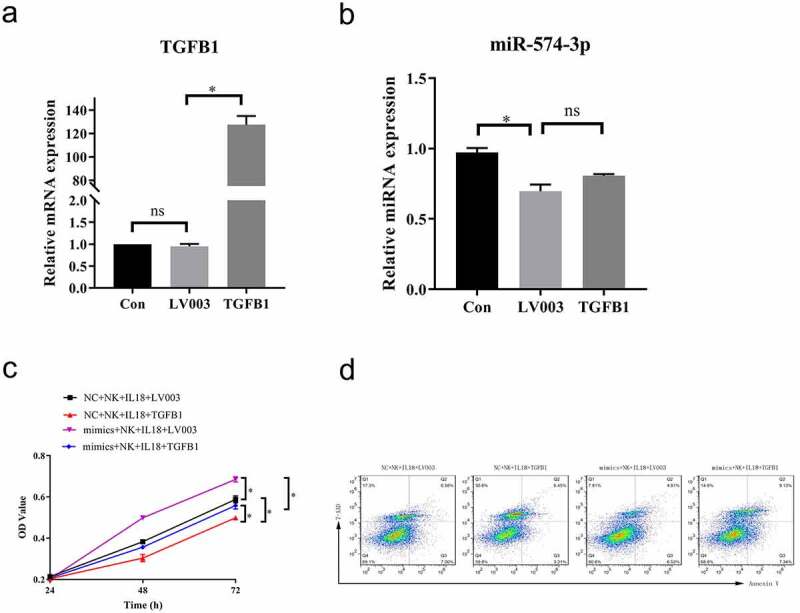
Overexpression of TGF-β1 rescued the effect of NK cells and IL-18

To test our hypothesis and verify the role of TGF-β1 in the antitumor effects of NK cells and IL-18 treatment, we overexpressed TGF-β1 in HCT116 (via transfection with LV003-TGF-β1 plasmid) to significantly increase levels of TGF-β1 in the LV003-TGF-β1 group compared with that in HCT116 cells only and in cells transfected with LV003 only (Figure 7a). However, overexpression of TGF-β1 did not alter the expression of miR-574-3p in overexpression TGF-β1 HCT116 cells compared to HCT116 cells trasfected with LV003 plasmid (Figure 7b). In coculture with NK cells and with IL-18 treatment, miR-574-3p overexpression significantly promoted cells proliferation and inhibited the rate of apoptosis rate (Figure 7c,d). Interestingly, when TGF-β1 was overexpressed, the function of miR-574-3p was reversed in both cells proliferation and apoptosis (Figure 7c,d). This further confirmed our hypothesis that NK cells and IL-18 exhibit antitumor effects by inhibiting the miR-574-3p/TGF-β1 axis.

a. Overexpression of TGF-β1 in HCT116 cells was confirmed via qPCR. b. qPCR results indicate that the expression of miR-574-3p was unperturbed when TGF-β1 was overexpressed compared with HCT116 cells tranfected with LV003 vector. c. MTS assays showing that TGF-β1 overexpression restored the proliferative promotional effect on HCT116 cells from miR-574-3p overexpression with NK cells coculture and IL-18 treatment. d. Representative fluorescence-activated cells sorting of 7-AAD-positive and annexin V-positive cells when TGF-β1 is overexpressed. TGF-β1 restored the inhibited apoptotic effect of HCT116 from miR-574-3p overexpression under NK cells and IL-18 treatment. NK: Natural killer; IL-18: Interleukin 18; MTS: 3-(4,5-Dimethylthiazol-2-yl)-5-(3-carboxymethoxyphenyl)-2-(4-sulfophenyl)-2 H-tetrazolium; qPCR: Quantitative polymerase chain reaction; TGF-β1: transforming growth factor beta 1. All data with error bars are presented as mean + SD, One-way analysis: * *p* < 0.05; ns: not significant.

## Discussion

IL-18 has a pivotal role in the regulation of the immune system and acceleration of immune response [4]. Specifically, IL-18 can activate NK cells to eliminate cancer cells and therefore is a potential target for cancer immunotherapy [6]. Because of the universal expression of IL-18 in various cell types throughout the gastrointestinal tract, we explored the potential antitumor effect of IL-18 in CRC and found that IL-18 can further activate NK cells to reduce cells proliferation and induce apoptosis in CRC. Therefore, future experiments should be designed to investigate the specific pathway of NK cells antitumor activity induced by IL-18 treatment.

miRNAs are small non-protein coding RNAs that are important post-transcriptional regulators in CRC [31]. More than 500 miRNA-target pairs have been discovered in CRC, although none of these have been developed for clinical use. As potential biomarkers, miRNAs are now of great interest to provide novel insights into CRC biology as well as potential therapies. In this research, through analyzing miRNA expression in HCT116 cells before and after coculturing with NK cells and IL-18 treatment, we identified five miRNAs that were affected by IL-18 treatment. First discovered in *C. elegans*, let-7c is one of the most well-known miRNAs in CRC [32]. Normally, let-7c is downregulated in CRC as well as in other types of cancers including pancreatic cancer, ovarian cancer, and breast cancer [3]. Previous research has linked let-7c to cells proliferation, cells cycle regulation, tumor cells migration, and chemoresistance. Specifically, downregulation of let-7c expression has been linked to poor prognosis in CRC patients, suggesting that let-7c functions as a tumor suppressor [33]. Triboulet et al. found that further downregulation of let-7c caused an increase of tumor incidence in mice [3], indicating that let-7c plays an important role in intestinal tumorigenesis in mice. Here, let-7c was further downregulated after coculturing with NK cells and treatment of IL-18. However, as the underlying regulatory mechanism of let-7c action with NK cells and IL-18 is unclear, an animal model is required to determine the effect of let-7c downregulation in vivo.

Increased expression of miR-574-3p in a human gastric cancer cell lines induced by TGF-β1 using PCR assays has been previously described [26]. This study by Zhang et al. also demonstrated that induction of miR-574-3p was specifically regulated by Smad4. Here when miR-574-3p was overexpressed, TGF-β1 expression was significantly downregulated, but there was no significant difference on miR-574-3p expression when TGF-β1 was overexpressed, indicating that there may be another regulatory pathway in HCT116 induced by coculturing with NK cells and treatment with IL-18. The SMAD4 signaling pathway is one of the most well-studied downstream pathways of miR-574-3p activity. Downregulation of miR-574-3p induced in osteosarcoma revealed that SMAD4, as a target of miR-574-3p, rescued cells from the tumor growth promoting effect of miR-574-3p [34]. In our study, IL-18 treatment combined with NK cells resulted in cells apoptosis and inhibited cells proliferation in vitro. Further, miRNA expression profiling following coculture with NK cells and treatment with IL-18 resulted in the significant downregulation of miR-574-3p expression. Similarly, Zhou et al. sequenced miRNAs obtained from 847 patients with gastric cancer and demonstrated the downregulation of miR-574-3p [25]. It has been reported that TGF-β1 can significantly induce miR-574-3p upregulation [25,26]. Zhang et al. demonstrated that Smad4 silencing significantly inhibited TGF-β1-induced miR-574-3p upregulation in AGS cells. Furthermore, they found that TGF-β1 significantly increased the activity of the dual-luciferase reporter that contains the Smad binding sites upstream of the miR-574 precursor sequence; these results suggest that the upregulation of miR-574-3p via TGF-β1 induction is functionally significant. In our study, the interaction between miR-574-3p and TGF-β1 was predicted via RNA sequencing and further proved by performing the dual-luciferase assay; therefore, we suggest that IL-18 promotes the antitumor ability of NK cells colcultured with HCT116 cells via the miR-574-3p/TGF-β1 axis. The results of our study have not yet been confirmed in other types of CRC cell lines and animal experiments, which remain a key in understanding the complex interactions between different components of the immune system and tumors. Additionally, whether IL-18 promotes the antitumor ability of T cells in CRC needs to be studied in detail in the future.

In this study, we demonstrated that IL-18 alone did not affect HCT116 cells but promoted the antitumor ability of NK cells in a coculture with HCT116 cells via the miR-574-3p/TGF-β1 axis; this finding suggests that IL-18 can be the new potential target for cancer immunotherapy for CRC.

## Highlights

1. IL-18 alone does not affect HCT116 cells.
2. IL-18 promotes the antitumor ability of NK cells in HCT116 cells.
3. miR-574-3p can inhibit the antitumor effects of IL-18-induced NK cells.
